# Recruiting males to the nursing profession: acceptability testing of the ‘Make a Difference with Nursing’ intervention for post-primary school students

**DOI:** 10.1186/s12912-022-00956-5

**Published:** 2022-07-04

**Authors:** Mark A. Linden, Gary Mitchell, Susan Carlisle, Debbie Rainey, Caroline Mulvenna, Catherine Monaghan

**Affiliations:** School of Nursing & Midwifery, Queen’s University Belfast, 97 Lisburn Road, Belfast, BT9 7BL UK

**Keywords:** Recruitment, Men, Post-Primary Schools, Education, Career, Nursing, Intervention

## Abstract

**Background:**

The nursing profession is facing a worldwide recruitment shortage which could be partially addressed by recruiting more males. However, in many developed countries numbers of male nurses have traditionally been low. To address this issue we developed and tested a post-primary school-based intervention to recruit more males into the nursing profession.

**Methods:**

Participants included thirty-four  female students drawn from an all-girls post-primary school, fifty-one males from an all-boys post-primary school and seven males and fifteen females drawn from a mixed gender post-primary school, all located in Northern Ireland. Participants were all sixteen years of age and were in receipt of careers education. The intervention titled ‘Make a Difference with Nursing’ was co-designed with careers teachers, nurses and post-primary school students. This video based intervention was created to fit within existing career lessons and featured nursing staff and students describing their experiences of the profession. Participants completed the six domains of Nursing as a Career Choice Questionnaire prior to viewing the intervention and again after viewing.

**Results:**

Positive and statistically significant increases in perceptions of the nursing profession were found between pre and post test scores across all six domains. No statistically significant differences in perceptions were found between males and females after watching the intervention. Comparison by school type showed statistically significant differences on the domains of prior healthcare exposure (*p* = 0.046; 95% CI -0.19 to 3.79) and job prospects (*p* = 0.005; 95% CI 1.04 to 7.01). Students from the all-girls school rated these more positively than those from the all-boys and mixed-gender schools.

**Conclusions:**

A short video presentation to post-primary school students is capable of changing how they perceive the nursing profession. Students expressed surprise over the variety of professional nursing roles suggesting that many were not well informed about the realities of nursing. Providing tailored resources for use in careers lessons can better educate students and teachers about nursing and provide positive portrayals of male role models in the profession.

## Introduction

The nursing profession faces an estimated global shortfall of approximately 9 million nurses by 2030 [1]. In the United Kingdom there are currently more than 40,000 nursing vacancies [2]. These shortages are leading to increased nurse workload, risk of error, adverse outcomes such as infection, sepsis or pneumonia and increased nurse burnout [3, 4]. Increasing the recruitment of men into the profession may provide a partial solution, however, traditionally this has proven challenging for a number of reasons.

In the UK, approximately 11% of nurses are male [5]. These numbers are similar to those found in the USA (9.4%;) [6] and Australia (12%) [7]. However, this is in contrast to countries that historically have higher percentages of men in nursing, for example Portugal (18%) [8], Iran (29%) [9] and Saudi Arabia (40%) [10].

Women comprise the vast majority of the profession which means it does not adequately represent the wider population [11]. The feminised nature of nursing may deter men from applying to degree programmes [12] and careers teachers/school counsellors who are uninformed may fail to offer it as an option to their male students [13].

Given the projected shortfall in nursing numbers, recruitment is an issue which has received a good deal of attention in the international literature. In Poland, a qualitative study involving 17 male nurses showed that vocation, interest in medicine, accident and pragmatism were cited as the main reason for their joining the profession [14]. In the UK, researchers have presented additional motivations including parental influence, caring, age and culture [15]. The same researchers also showed a number of demotivating factors which included exclusion from gender-specific areas of care and issues of sexuality and identity. Further work has suggested job security, income, prestige and acquiring transferrable knowledge and skills as being important [14, 16, 17]. Others have suggested a number of systemic and gender specific barriers facing men including; lack of course content relating to men; nurses being referred to as ‘she’; no active male recruitment; a fear of suspect touch in working with female patients; lack of male role models [11].

The role of significant others in influencing whether students chose a career in nursing was investigated by a team of UK researchers [18]. Employing essay style questions, the researchers asked 68 first year undergraduate nursing students (*n* = 61 women) about their reasons for joining the profession and what role, if any, their family or friends had in their decision making. Family members within the nursing profession were seen to offer a realistic perspective, however some parents were reported as perceiving nursing as low status. Findings suggested friends were a source of support as were previous interactions with nurses and encouragement of teachers [18]. While responses were primarily from women this study demonstrates the importance of relationships in guiding students to consider nursing as a career. Careers teachers are well placed to discuss future job prospects with students but need to be better informed about the profession and offer it as an option to male students.

Researchers have also explored the reasons men remain in the profession. Experienced male practitioners in the US chose to stay in the profession due to early life experiences, positive working experiences, and a sense of belonging which allowed them to overcome the perceived stigma of being a man in nursing [19]. Men also tend to seek more senior nursing positions which result in them achieving higher salaries [20]. A recent qualitative evidence synthesis around motivating factors that led men to undertake nursing reported that early exposure to nursing and other healthcare professionals were key influencing factors [21]. Poor public understanding and perceptions about the nursing profession have likely contributed to lower uptake of nursing amongst men in some countries.This may be attributed to the perception that nursing is ‘women's work’ [12].Evidence also suggests that once enrolled on a professional nursing programme, male students are less likely to complete their programme when compared to female students [22]. Stigma experienced by men who choose a career in nursing remains a challenge. Qualitative research undertaken in Spain illustrated how male nurses often experienced discomfort when patients and family members expected nursing care to be delivered by a female [23]. It would seem we now know many of factors, barriers and facilitators as to why men enter the profession but require greater efforts in their recruitment.

### Study aim and hypotheses

This study aimed to co-design an intervention which would fit within the existing school curriculum for use within careers lessons. The primary focus of this was to inform boys about the nursing profession and directly address the issue of male recruitment. However, we were conscious of not wanting to dissuade girls from considering nursing and took this into account by including representation from both female and male nursing staff and students. The presentation of positive male and female role models was intended to bring about change in how young men and women perceived the nursing profession. We first hypothesised that there would be a statistically significant difference between pre and post-test perceptions of the profession following viewing of the intervention. Secondly, we hypothesised that there would be differences between girls and boys and lastly that there would be differences depending on which type of post-primary school students attended.

## Methods

### Study design

The study utilised a pre-post-test design to test for the effects of the intervention on post-primary school student perceptions of the nursing profession. The independent variables included pre and post test scores on student perceptions, gender at two levels (female and male) and school type at three levels (all-girls school, all-boys schools and mixed gender school). The dependent variable was perception of the nursing profession measured using the Nursing as a Career Choice Questionnaire [24].

### Participants

Al participants were sixteen years of age and were taking part in regular careers lessons. The purpose of these lessons was to give students information, guidance and support from their careers teachers to decide on future plans for choosing an occupation. Three post-primary schools agreed to take part comprising an all-girls, all-boys and an integrated or mixed gender school. One hundred and twenty-seven students completed the first questionnaire while one hundred and seventeen completed the second. When pre and post questionnaires were matched, complete data were available for one hundred and seven participants. Thirty-four girls took part from the all-girls school, fifty-one males from the all-boys school and fifteen girls and seven boys from the mixed-gender school.

### The intervention

The ‘Make a Difference with Nursing’ intervention was co-designed with careers teachers, nurses, post-primary school students and members of the research team over a period of approximately one year (2020–2021). Working with an experienced video production company the group met on four occasions to discuss format, content and main messages. Current staff and students were recruited to talk about their experience of training, the degree programme at the authors institution and the nursing profession. The intervention featured six nursing students (3 females and 3 males) in addition to one male and one female member of staff and had a duration of 20 min and 41 s. In order to provide additional information, the intervention also included some facts and figures, portrayed through graphics, about the profession and the degree programme at the authors institution. These included the duration of the BSc degree programme, entry requirements, pay scales post qualification and nursing core values as identified by the Northern Ireland Practice and Education Council for Nursing and Midwifery (https://nipec.hscni.net/). We wanted the intervention to have an active component to better engage students with content and make them consider their perceptions of the nursing profession. Three discussion sections were added to the video where careers teachers could pause the content and allow time for students to answer 3–4 questions. Discussion topics included myths about the nursing profession, why men and women should be nurses and the factors that made a good nurse. The completed intervention can be viewed here https://youtu.be/Xk9qph4Y8F4.

### Measures

The Nursing as a Career Choice Questionnaire [24] is a 35 item psychometrically validated tool which assesses respondents views on six factors including personal interest, prior healthcare exposure, self-efficacy, perceived nature of work, job prospects and social influences. Participants are asked to respond on a five point Likert scale ranging from ‘1 = Strongly disagree’ to ‘5 = Strongly agree’. Internal consistency for the scale employing Cronbach’s alpha was 0.94 with test–retest reliability of 0.60 [24]. Two online questionnaires were used to assess pre and post-test perceptions. Questionnaire one contained the Nursing as a Career Choice Questionnaire as did questionnaire two. However, questionnaire two also included some questions to explore the acceptability and impact of the intervention. Additional questions included ‘Do you think young people of your age in other Schools would benefit from watching this video?’ and ‘Would you want to learn more about nursing after watching the video?’.

### Ethical approval

This study was reviewed and approved by a University ethics committee (Ref:MHLS21_102). Participants and their parents were provided with information about the study which included issues of confidentiality, right of withdrawal and data protection. Participants were required to indicate their consent to take part by ticking a series of boxes in response to questions contained at the start of the first online questionnaire. Participants received a £10 gift certificate in thanks for their completion of both questionnaires.

### Data collection

Data collection took place between October–November 2021. Employing convenience sampling, post-primary school students, in receipt of careers education, from three schools in Northern Ireland were invited to take part. Permission was sought from the principals of these schools who connected the research team with careers teachers. Careers teachers emailed letters of invitation to parents and students aged 16 years to inform them that the study was taking place. One week later, letters of information were sent to parents and students. Students were given the option to opt out of the research by informing their careers teacher. Those students who were interested in taking part were then emailed a link to the first of two Microsoft Forms online questionnaires. Approximately one week later students viewed the ‘Make a Difference with Nursing’ video and completed the second questionnaire.

### Statistical analysis

Data were analysed using IBM SPSS Statistics version 27 for windows with 20% of data entry checked by a second member of research team to ensure rigor. Descriptive statistics were used to summarise participants’ data and check for normality. The dependent t-test was used to explore pre and post-test differences after viewing the intervention, while independent t-tests were used to explore gender differences. Analysis of variance (ANOVA), with planned comparisons, tested the effect of School type on perceptions of the nursing profession.

## Results

In response to the question ‘Do you think young people of your age in other Schools would benefit from watching this video?’ 84 (78.5%) students said ‘yes’, 19 (17.8%) said ‘maybe’ and 3 (2.8%) said ‘no’. When asked whether the video made students want to learn more about the profession, 42 (39.3%) said ‘yes’, 48 (44.9%) said maybe and 17 (15.9%) said ‘no’.

### Pre and post intervention changes in perceptions

Dependent t-tests showed that the intervention increased student perceptions about the nursing profession across all six domains of the questionnaire. These differences were statistically significant at the *p* < 0.01 level for self-efficacy and social influences and *p* < 0.001 level for personal interest, prior healthcare exposure, perceived nature of the work and job prospects. Table 1 shows the means, standard deviations, 95%CIs and effect size for these tests. Positive improvements in perceptions were found across all domains, however, these were greatest in relation to job prospects [t(106) = -4.72, *p* < 0.001; mean difference = 2.18, Cohen’s d = 4.79] and prior healthcare exposure [t(106) = -6.47, *p* < 0.001; mean difference = 1.95, Cohen’s d = 3.12].

**Table 1:** Pre and post intervention measures of student perceptions

	Pre Mean(SD)	Post Mean(SD)	95%CIs	Cohen’s d
Personal interest	20.21(2.27)	21.61(2.30)	-1.83 to -0.95**	2.29
Prior healthcare exposure	19.72(2.88)	21.67(3.31)	-2.55 to -1.35**	3.12
Self-efficacy	11.99(2.96)	12.81(2.92)	-1.37 to -0.28*	2.85
Perceived nature of work	17.34(3.26)	18.76(3.15)	-1.89 to -0.95**	2.45
Job prospects	22.35(4.12)	24.53(4.96)	-3.10 to -1.27**	4.79
Social influences	25.05(4.80)	26.30(4.93)	-2.17 to -0.34*	4.78

^*^p < 0.01, **p < 0.001

### Gender differences in student perceptions

Independent t-tests were conducted to test the hypothesis that gender differences would influence student perceptions of the profession after viewing the intervention. Matched data from questionnaire one were subtracted from that of questionnaire two to produce a single dependent variable for each individual across the six domains. Results showed no statistically significant differences based on how boys or girls perceived the nursing profession after taking part in the intervention.

### Impact of school type on student perceptions

Between subjects ANOVAs sought to test the hypothesis that differences would exist on student perceptions based on school type (all-girls, all-boys and mixed-gender). No statistically significant differences were found for the domains of personal interest, self-efficacy, perceived nature of the work or social influences. However, statistically significant differences were found on the domains of prior healthcare exposure [F(2, 104) = 3.168, *p *= 0.046, patrial eta squared = 0.057] and job prospects [F(2, 104) = 5.662, *p* = 0.005 patrial eta squared = 0.098]. Planned comparisons using Tukey’s HSD showed that these differences lay between the all-girls school and both the all-boys (*p* = 0.041, 95%CI 0.08 to 4.92) and mixed-gender schools (*p* = 0.005, 95%CI 1.04 to 7.01) for job prospects. Students from the all-girls school rated job prospects higher (mean = 4.21) after watching the intervention than students from the all-boys (mean = 1.71) or mixed-gender schools (mean = 0.18). Planned comparison showed no statistically significant differences between school types for prior healthcare exposure. However, *p* values did approach significance between the all-girls school and both the all-boys (*p* = 0.078, 95% CI -0.13 to 3.09) and mixed-gender schools (*p* = 0.084, 95% CI -0.19 to 3.79). Again, students from the all-girls school (mean = 3.03) rated changes in perceptions of prior healthcare exposure more highly than the all-boys (mean = 1.55) and mixed-gender (mean = 1.23) schools after viewing the intervention. See Table 2 for means and standard deviations (SD) for pre and post perceptions scores by school type.

**Table 2:** Pre and post intervention perceptions by school type

	All-Girls Mean(SD) *N* = 34	All-Boys Mean(SD) *N* = 51	Integrated Mean(SD) *N* = 22
Personal interest 1	20.09 (2.48)	19.82 (1.81)	21.32 (2.61)
Personal interest 2	21.68 (2.36)	21.33 (2.19)	22.14 (2.45)
Prior healthcare exposure 1	19.82 (2.70)	19.25 (3.05)	20.64 (2.63)
Prior healthcare exposure 2	22.85 (3.86)	20.80 (2.66)	21.86 (3.31)
Self-efficacy 1	12.24 (3.33)	11.88 (2.80)	11.86 (2.83)
Self-efficacy 2	13.41 (3.51)	12.49 (2.60)	12.64 (2.57)
Perceived nature of work 1	18.18 (3.12)	16.71 (3.07)	17.50 (3.71)
Perceived nature of work 2	19.74 (2.77)	18.39 (3.14)	18.09 (3.50)
Job prospects 1	21.32 (4.39)	22.84 (3.96)	22.77 (3.96)
Job prospects 2	25.53 (5.44)	24.55 (4.55)	22.95 (4.91)
Social influences 1	24.97 (4.81)	24.55 (4.34)	26.32 (5.73)
Social influences 2	27.53 (4.00)	25.51 (4.74)	26.23 (6.34)

## Discussion

This manuscript describes a newly co-designed intervention intended to recruit more males to the nursing profession. Findings were positive and showed pre and post-test differences indicating the intervention had brought about changes in how students perceived the nursing profession. All of these changes increased, demonstrating more positive perceptions. Previous recruitment campaigns have sought to encourage men by portraying male nurses as technologically capable and macho [25]. Our approach was to provide real life role models who were relatable and open about their choice of nursing. This honest portrayal seemed to resonate with students in the study and may have altered their perceptions.

When male and female student perceptions were compared results showed no statistically significant difference. We interpret this finding as meaning that the intervention had a similar effect on male and female student perceptions. This is an important finding as it suggests that changes in perceptions brought about by the intervention were not influenced by student gender. Male students held more positive perceptions after viewing the intervention. Evidence suggests that public perceptions of nursing are positive, and yet there remains a global shortfall in recruitment [26]. The feminised nature of nursing may deter careers teachers from offering it as a potential occupation to their male students [27]. Our intervention provides targeted information to students and careers teachers while also providing positive male role models for potential recruits. We must note that while a positive change in male perceptions found in this study is encouraging we do not yet know whether this will transfer into future applications to nursing programmes.

Whilst four out of six domains did not show a difference based on school type, two did. The lack of differences shows that school type did not influence student perceptions in relation to personal interest, self-efficacy, perceived nature of work, and social influences. These findings are encouraging and shows that the intervention was able to increase interest in a career in nursing irrespective of school type. However, differences were found in student perceptions for the domains of prior healthcare exposure and job prospects. Students from the all-girls school rated these domains more positively than those from the all-boys and integrated gender schools.

‘Prior healthcare exposure’ refers to previous positive or negative experiences of nursing which could influence the decision to pursue a career. Questions in this domain include taking care of a family member and being taken care of by a nurse but also include the influence of significant others. It is possible that the tacit endorsement of the intervention by their careers teacher influenced students from the all-girls school to a greater extent than that of the other schools. Researchers have shown that family, friends and teachers all have a role to play in decisions around joining the nursing profession [18].

The domain of ‘job prospects’ refers to perceptions around income, employment opportunities and job stability. It has been suggested that men’s career choice is influenced by hegemonic masculinity which relates to authority, heterosexuality, power and money [28]. Indeed, one of the reasons male nurses choose to leave the profession is financial [14]. If male students felt that renumeration for a career in nursing was below their expectations they may have rated ‘job prospects’ as lower in comparison to students from the all-girls school.

‘Make a Difference with Nursing’ is a tailor made educational intervention for students which focuses on a single Nursing degree programme. However, many of the portrayals of male nursing students would be readily transferred to nursing programmes around the world. As such, our intervention could be viewed as a model for other schools of nursing who wished to encourage more male applicants.

### Limitations

We utilised a self-report measures of perceptions of the nursing profession which is clearly open to social desirability bias. However, students had no contact with members of the research team which may have reduced such bias. It was unfortunate that only 22 students from the integrated or mixed gender school chose to take part in the research. This was the smallest of the three groups and may mean that the school ethos had less of an impact on findings than may have been expected. Finally, the intervention referred to a single nursing degree programme offered in one university in the UK. Therefore the generalisability of this work may be in question. It is hoped that this project can be used as an easily replicable model for other degree programmes. It may be possible to create a national or international version of ‘Make a Difference with Nursing’, however, variability in degree programmes at the international level may preclude this. While improving perceptions of the profession is an important outcome of this work it is uncertain whether this will result in future applications to degree programmes.

### Future research

It would be important to monitor the future uptake and impact of this intervention. For example, how many schools use it in careers lessons and is there an increase in applications to nursing degree programmes from males. This will require longitudinal monitoring of admissions data which is routinely collected. It would also be useful to ask students who apply to our nursing programme whether they had viewed the current intervention.

It would be possible to undertake a larger scale trial of the intervention which included many more schools. This would increase the strength of the evidence for its use and also mean that many more pupils would be given the opportunity to see the intervention.

Bringing together a number of institutions across the UK to create a similar programme would be an achievable future goal. Nursing degree programmes may have negligible regional differences in how they are operationalised, however, the core principles and nursing qualities identified through the NMC are the same. A UK wide programme would have advantages in terms of scale and reach and has the potential to encourage more applications from males at the national level.

Any nursing programme could tailor use of our intervention for their own institution. We used current staff and students who volunteered their time to talk enthusiastically about nursing and provide information about the degree programme. This was the main element of the intervention which could be easily replicated elsewhere. However, any such efforts should be co-developed with careers teachers and pupils to ensure it is relevant to their needs. As with any technology, these interventions should not remain static and need to be updated as nursing programmes change and to ensure the intervention appears contemporary to pupils.

## Conclusions

The intervention positively brought about changes in student perceptions of the nursing profession, irrespective of gender. As an educational tool the intervention sought to inform primarily students, but also their careers teachers about the profession. The provision of accurate and up to date information which portrays modern nursing can help dispel misunderstanding and change student perceptions of the profession. It is hoped that by improving teacher and student perceptions that more young men may be introduced to the rewards of nursing and consider it as a career choice.

## Data Availability

The authors confirm that the data supporting the findings of this study are available upon request made to the corresponding author.
